# Cardiac dysfunction from cancer and cancer therapy: new pathways for the prevention of late cardiotoxicity

**DOI:** 10.1007/s00395-021-00903-6

**Published:** 2021-10-20

**Authors:** Lars Michel, Matthias Totzeck, Tienush Rassaf

**Affiliations:** Department of Cardiology and Vascular Medicine, West German Heart and Vascular Center, University Hospital Essen, Hufelandstraße 55, 45147 Essen, Germany

This editorial refers to ‘Anthracycline-free tumor elimination in mice leads to functional and molecular cardiac recovery from cancer-induced alterations in contrast to long-lasting doxorubicin treatment effects’, by Pietzsch et al.

While the toxic effects of common cancer therapies on the cardiovascular system are increasingly understood mechanistically, the precise direct effects of cancers on cardiovascular diseases remain incompletely resolved [6, 18, 24]. This can in part be related to two major problems: (i) in clinical routine, cancer-related effects can hardly be separated from cancer therapy-induced cardiotoxicity, and (ii) many preclinical studies lack rigorous cancer models and cardiotoxicity is frequently tested in healthy specimens. As cancer itself may pose a major burden to cardiovascular health with significant consequences on cardiovascular outcomes, the understanding of the underlying mechanisms is crucial for a comprehensive understanding of the biology.

Advanced cancer is commonly associated with cardiac atrophy, leading to myocardial structural alterations, cardiac metabolism and myocardial remodelling. A decline in skeletal muscle mass is paralleled by a rarefication of the contractile apparatus in myocardial tissue with decreased gene expression of troponin I, associated with increased fibrosis and a reduced functional capacity, as reflected by decreased fractional shortening and cardiac output [3, 22]. Tumor-associated factors were shown to induce changes in cardiac redox homeostasis resulting in higher levels of reactive oxygen species, reduced mitophagy, and lower hypoxia resistance [9]. Glucose metabolism appears to exhibit a further key element of cancer-induced cardiomyopathy. Low insulin plasma levels in cancer cachexia lead to reduced glucose uptake in cardiac tissue and promote cardiac atrophy and subsequent systolic heart failure. In skeletal muscle, an increased insulin resistance is found, and skeletal muscle degradation provides energy substrates through proteolysis that in part promote hepatic gluconeogenesis [3]. In parallel, cardiac triglyceride levels decrease in models for advanced cancers, while CD36 (fatty acid translocase) on cardiomyocytes is upregulated [21]. Systemic effects of cancer on multiple signalling pathways were, furthermore, identified. Exemplarily, cancer alters the growth factor-regulated phosphoinositide 3-kinase (PI3K)-AKT pathway [7] that is also profoundly involved in myocardial signalling with diverse downstream effects [5].

Of note, growing evidence adds another dimension to the diverse coherences of heart and cancer, as cardiovascular disease may also accelerate tumor growth. Preclinical findings indicate that heart failure may promote progression of cancer, as seen in a heart failure mouse model that indicated a correlation between worsening left ventricular function and tumor growth. A similar effect was recapitulated in a transverse aortic constriction mouse model, showing that cardiac hypertrophy/remodelling in the absence of systolic heart failure induced accelerated tumor growth. Several inflammatory factors secreted by the myocardium including periostin and serpinA3 were proposed to promote tumor growth, as pro-inflammatory biomarkers correlate with new-onset cancer in humans [1, 11].

Cardiac cachexia negatively impacts morbidity and mortality of patients at risk, and effective therapies have not yet been identified [10]. In cancer patients, cachexia with potential adverse consequences for cardiac integrity affects up to 74% of cancer patients, with significant consequences on long-term cardiovascular health [3]. Understanding the underlying pathomechanisms and their implications on acute and late cardiac dysfunction is crucial to optimize the management of cardio-oncology patients [6, 15, 18, 23].

**Fig. 1:**
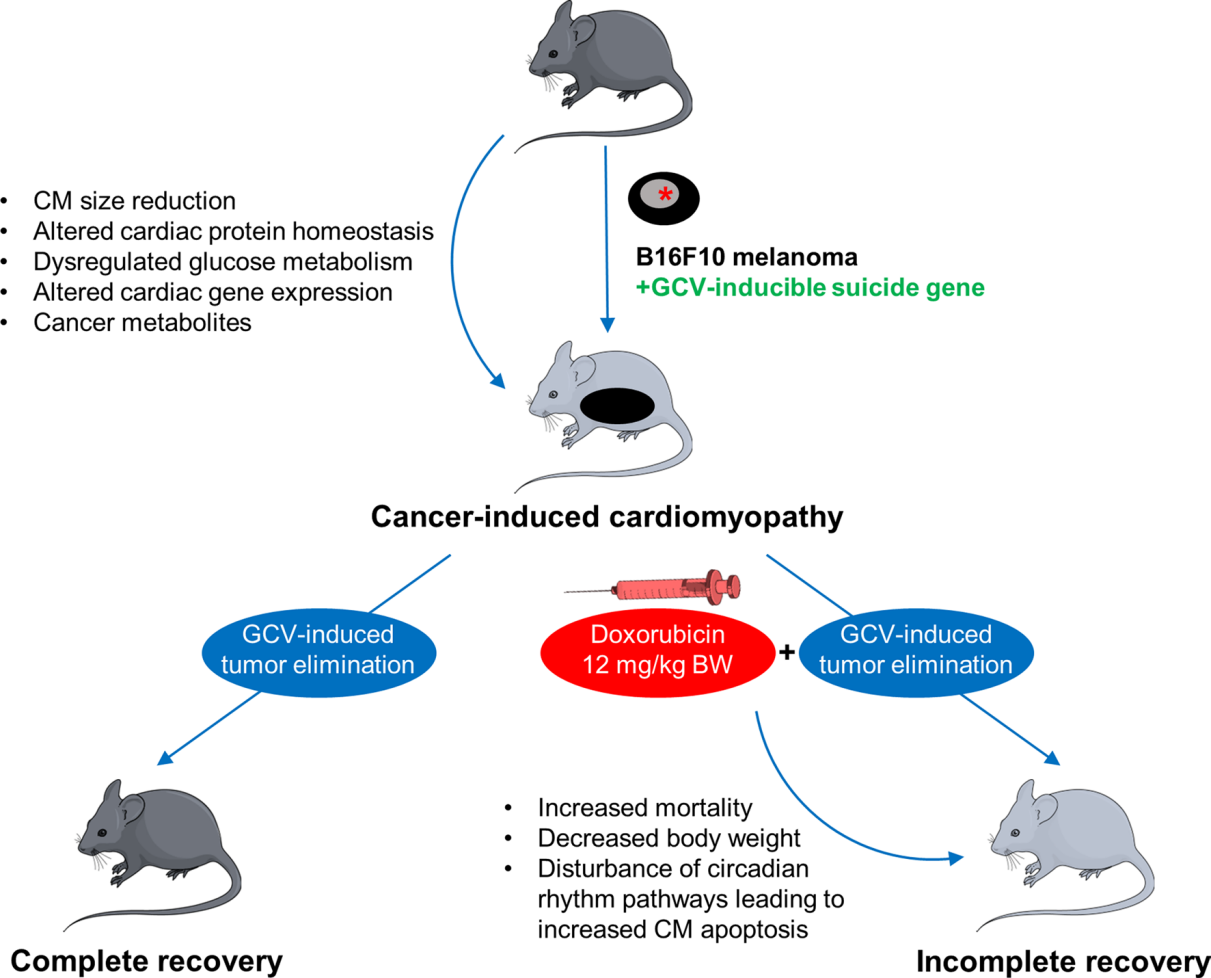
Recovery from cancer-induced cardiomyopathy. Scheme of cancer-related cardiomyopathy in the reversible B16F10 transgenic melanoma mouse model and its recovery in the absence and presence of doxorubicin therapy. *BW* body weight, *CM* cardiomyocytes, *GCV* ganciclovir

In this issue of Basic Research in Cardiology, Pietzsch and colleagues shed new light on the often neglected impact of the underlying cancer on the recovery after cancer-induced cardiomyopathy [16]. As a novel approach, the authors establish a reversible melanoma mouse model on the basis of the common B16F10 melanoma cell line that was genetically modified via lentiviral transduction of a suicide gene that could induce cell death upon exposure to ganciclovir. With this, the authors were able to observe the effects of cancer on cardiac integrity and its subsequent elimination independently from a specific cancer therapy. To elaborate on therapy-related effects, the authors compared these mice to specimens that were treated with doxorubicin as the prototype of cardiotoxic cancer therapy. Finally, molecular findings were confirmed ex vivo in neonatal rat cardiomyocytes (Fig. 1).

With their in vivo approach, the authors determined that cancer-related cardiomyopathy was fully reversible after therapy-free tumor elimination. Several key metabolic and functional pathways that were shown to deteriorate during advanced cancer including glucose metabolism (GLUT1 and GLUT4 expression, plasma insulin), lipid metabolism (cardiac expression of CD36) and gene expression profiles normalized after a recovery period of 70 ± 5 days. Biochemical observations were paralleled by recovered physical strength, a physiological response to angiotensin II exposure and normalized functional parameters. Strikingly, mice that received doxorubicin treatment did not show complete recovery despite tumor elimination. Particularly, mice showed sustained changes in cardiac gene expression patterns that particularly involved circadian rhythm genes, such as *Bmal1*, *Clock*, *Period1-3*, and *Cryptochrome 1/2*. To determine the effect of this, *Bmal1* was silenced by si-RNA in neonatal rat cardiomyocyte culture, which lead to a reduced mitochondrial membrane potential and increased release of cytochrome C, leading to an increased rate of apoptosis in affected cells. The authors hereby concluded that the observed changes of circadian rhythm genes exhibited significant effects on cardiomyocyte integrity and that its disruption by doxorubicin therapy can promote cardiomyocyte apoptosis, leading to a persisting disruption of cardiac integrity in contrast to the otherwise fully reversible effects of cancer on the heart.

Pietzsch and colleagues now propose that cancer induces substantial functional, metabolic, and inflammatory modifications, which may be reversible or serve as a potential therapeutic target. This is meticulously assessed for anthracyclines by the authors. Currently, cardiac long-term effects and their recovery from cancer itself lack a clinical demonstration and play a subordinate role in the risk assessment of patients, while therapy-related effects are extensively characterized and included in cardiac risk assessment. Arguably, a mechanistic separation of cancer- and cancer therapy-related effects cannot be easily achieved for all forms of cancer therapies. In a collective of lung cancer patients receiving ICI therapy, more frequent severe immune-related adverse events were found in patients with high tumor burden (odds ratio 8.62) [19], and a comparable observation was demonstrated in a melanoma mouse model, where early cardiotoxicity from ICI therapy in the form of left ventricular dysfunction was only found in tumor-bearing mice [12, 14]. In CAR-T cell therapy, cytokine release syndrome (CRS) often leads to adverse cardiovascular reactions. The severity of CRS is difficult to predict in individual patients, and tumor burden is a main risk factor that is associated with severe CRS [4, 8]. Hence, presence of a tumor and the overall tumor burden will likely impact toxicity in these modern forms of oncological treatment.

Circadian rhythm has been a largely neglected factor in both cardio-oncology and cardiovascular medicine in general [2, 17]. The authors identify distinct persisting changes in cardiac circadian rhythm pathways only upon exposure to doxorubicin, including downregulation of the core clock protein BMAL1. While this association was already proposed before [2], the authors go further by demonstrating that silencing of *Bmal1* induces elevated apoptosis ex vivo, thereby proving a causative role of circadian rhythm signalling in the development of cancer therapy-related cardiotoxicity. It remains to be said that it is still not fully understood so far to what extent cancer itself influences the degree of cardiotoxicity caused by anthracyclines, since a comparison of treated mice with cancer to a control group of mice without cancer is crucial. The next developments in the field of basic science on the mechanisms of chemotherapy-associated cardiotoxicity can be eagerly awaited here.

Finally, the practical relevance for the clinical work remains, as consequences cannot be drawn based on the basis of the new data from the present study. Decreased expression of GLUT1 and GLUT4 together with lowered insulin levels corroborate the important role of glucose metabolism in maintaining cardiac integrity in cancer patients. It remains elusive whether monitoring of biomarkers, e.g., plasma insulin/c-peptide may serve as a novel risk factor [13]. Pathological findings may, however, provide new therapeutic avenues for medical and non-medical interventions including insulin supplementation as proposed before or exercise therapy, considering its substantial role on glucose metabolism in patients [20, 21]. Undoubtedly, the findings show a great potential for future research aiming to gain a broader understanding of the complex mechanisms behind cardiotoxicity from cancer and cancer therapy.
